# Machine learning-based discovery of UPP1 as a key oncogene in tumorigenesis and immune escape in gliomas

**DOI:** 10.3389/fimmu.2024.1475206

**Published:** 2024-09-24

**Authors:** Zigui Chen, Chao Liu, Chunyuan Zhang, Ying Xia, Jun Peng, Changfeng Miao, Qisheng Luo

**Affiliations:** ^1^ Department of Neurosurgery, Haikou Affiliated Hospital of Central South University Xiangya School of Medicine, Haikou, China; ^2^ Department of Neurosurgery, Central Hospital of Zhuzhou, Zhuzhou, Hunan, China; ^3^ Department of Neurosurgery, Affiliated Hospital of Youjiang Medical University for Nationalities, Baise, Guangxi, China; ^4^ Guangxi Engineering Research Center for Biomaterials in Bone and Joint Degenerative Diseases, Baise, Guangxi, China; ^5^ Department of Neurosurgery Second Branche, Hunan Provincial People’s Hospital (The First Affiliated Hospital of Hunan Normal University), Changsha, Hunan, China

**Keywords:** UPP1, glioma, immunotherapy, machine learning, single-cell sequencing

## Abstract

**Introduction:**

Gliomas are the most common and aggressive type of primary brain tumor, with a poor prognosis despite current treatment approaches. Understanding the molecular mechanisms underlying glioma development and progression is critical for improving therapies and patient outcomes.

**Methods:**

The current study comprehensively analyzed large-scale single-cell RNA sequencing and bulk RNA sequencing of glioma samples. By utilizing a series of advanced computational methods, this integrative approach identified the gene UPP1 (Uridine Phosphorylase 1) as a novel driver of glioma tumorigenesis and immune evasion.

**Results:**

High levels of UPP1 were linked to poor survival rates in patients. Functional experiments demonstrated that UPP1 promotes tumor cell proliferation and invasion and suppresses anti-tumor immune responses. Moreover, UPP1 was found to be an effective predictor of mutation patterns, drug response, immunotherapy effectiveness, and immune characteristics.

**Conclusions:**

These findings highlight the power of combining diverse machine learning methods to identify valuable clinical markers involved in glioma pathogenesis. Identifying UPP1 as a tumor growth and immune escape driver may be a promising therapeutic target for this devastating disease.

## Introduction

Gliomas are the most common and aggressive type of primary brain tumor, with a poor prognosis despite current treatment approaches. Understanding the molecular mechanisms underlying glioma development and progression is critical for improving therapies and patient outcomes (1). Recent advances in single-cell sequencing (scRNA-seq) have provided unprecedented resolution into the cellular heterogeneity of gliomas, revealing diverse populations of tumor, immune, and stromal cells (2, 3). At the same time, bulk tumor sequencing has identified key driver mutations and signaling pathways dysregulated in gliomas (4, 5). However, integrating single-cell and bulk tumor data to identify critical genes and pathways remains an important challenge.

Immune evasion is a key hallmark of cancer, where tumor cells are able to avoid detection and destruction by the body’s immune system. Understanding the mechanisms of immune evasion in cancer is crucial for the development of effective immunotherapies, which aim to overcome these immune evasion strategies and reactivate the body’s immune system to recognize and eliminate cancer cells (6).

In this study, we utilized diverse machine learning methods to comprehensively analyze scRNA-seq and bulk RNA sequencing of glioma samples (7). Through a series of advanced computational techniques, this integrative approach identified UPP1 (Uridine Phosphorylase 1) as a novel driver of glioma tumorigenesis and immune evasion. High UPP1 expression was linked to poor patient survival. Functional experiments revealed that UPP1 promotes tumor cell proliferation and invasion while suppressing anti-tumor immune responses. Additionally, UPP1 effectively predicted mutation characteristics, drug response, immunotherapy response, and immune features. These findings highlight the power of integrating single-cell and bulk tumor data from over 3,000 samples to identify critical genes involved in glioma pathogenesis. Identifying UPP1 as a tumor growth and immune escape driver suggests it may be a promising therapeutic target for this devastating disease.

## Materials and methods

### Data collection and processing

The scRNA-seq data of human glioblastoma (GBM) samples were obtained from the Single Cell Portal platform (SCP50 and SCP393) and processed using Smart-seq2. The bulk-sequencing data of human glioma samples were obtained from the TCGA (The Cancer Genome Atlas), CGGA (Chinese Glioma Genome Atlas), and GEO (Gene Expression Omnibus) databases. The current study included over 3,000 samples. The raw data from the GEO database was generated using the Affymetrix and Agilent platforms. The robust multichip average (RMA) technique accomplished the background correction and normalization. The RNA-sequencing data were obtained from the TCGA and CGGA data sites. Transcripts per kilobase million (TPM) values were created by converting the fragments per kilobase million (FPKM) values into values with a signal strength comparable to the RMA-processed values.

### Computational analysis

Uniform Manifold Approximation and Projection (UMAP) function from the R package Seurat was used to depict the microenvironment cells in the scRNA-seq data. Differentially expressed genes (DEGs) between the immune cells and neoplastic cells were identified. 182 immune escape (IE) pathway genes were collected (8). Weighted Correlation Network Analysis (WGCNA) was performed on the TCGA glioma dataset to determine the IE-related genes (9). Soft threshold settings were established to ensure a scale-free topology network and generate a TOM matrix. A power of β = 10 was used as the parameter. Blue module genes were extracted for subsequent analysis. The intersected genes between IE-related genes and DEGs were identified. Univariate Cox regression analysis was performed on intersected genes. Machine learning, RSF (Random Survival Forests) analysis (10), was performed on prognostic intersected genes. Machine learning, LASSO (Least Absolute Shrinkage and Selection Operator) regression analysis (11), was further performed on prognostic intersected genes. The R package survminer was used to create the survival curves of UPP1-related groups. Gene Set Enrichment Analysis (GSEA) was performed on UPP1. The R package oncoPredict was used to predict drug responses related to UPP1 (12). GISTIC 2.0 analysis was performed on UPP1 (13). The R packages maftools was used to generate the mutation landscape (14). The R package ComplexHeatmap was used to generate a heatmap of the immune infiltrating cells calculated by TIMER, MCPcounter, and ssGSEA (15–18) related to UPP1. The R package ComplexHeatmap was also to create a heatmap of the immune modulators related to UPP1.

### *In vitro* validation on UPP1

The glioma cell lines U251 and LN229 and microglia cell line HMC3 were purchased from iCell. Two siRNA sequences of UPP1 (Forward AGGCAGAGUUUGAGCAGAUTT; Forward UCAAGAAGAAACUGAGCAATT) were used to silence the expression of UPP1. Total RNA was extracted from siRNA-transfected glioma cells. The extracted RNA was then reverse-transcribed into cDNA using a reverse transcriptase enzyme. Next, the cDNA was used as a qPCR amplification template. Gene-specific primers were used to measure the abundance of target gene transcripts. The qPCR reaction was monitored in real-time, allowing for precise quantification of mRNA levels. Relative expression was calculated using the 2^-ΔΔCt method, with normalization to endogenous control genes. The EdU assay was employed to assess cell proliferation. Glioma cells were incubated with the thymidine analog EdU, which gets incorporated into the DNA of proliferating cells during S-phase. The stained cells were then analyzed by microscopy. The percentage of EdU-positive cells reflects the fraction of proliferating cells in the population, providing a quantitative measure of cell proliferation. The Transwell assay was used to evaluate the migratory capabilities of glioma cells. Cells were seeded onto the top chamber of a Transwell plate with a porous membrane. Cells that migrated through the porous membrane to the bottom chamber were quantified. The Co-culture Transwell assay was used to evaluate the migratory capabilities of macrophages. Macrophages were seeded onto the top chamber of a Transwell plate, and glioma cells were seeded onto the down chamber of a Transwell plate. Cells that migrated through the porous membrane to the bottom chamber were quantified.

### Statistical analysis

All statistical analyses were conducted with R. Student’s t-test and wilcoxon test were used to compare normally distributed variables and non-normally distributed data between the two groups, respectively. P <0.05 was considered statistically significant.

## Results

### The scRNA-seq analysis for malignant markers

**Figure 1:**
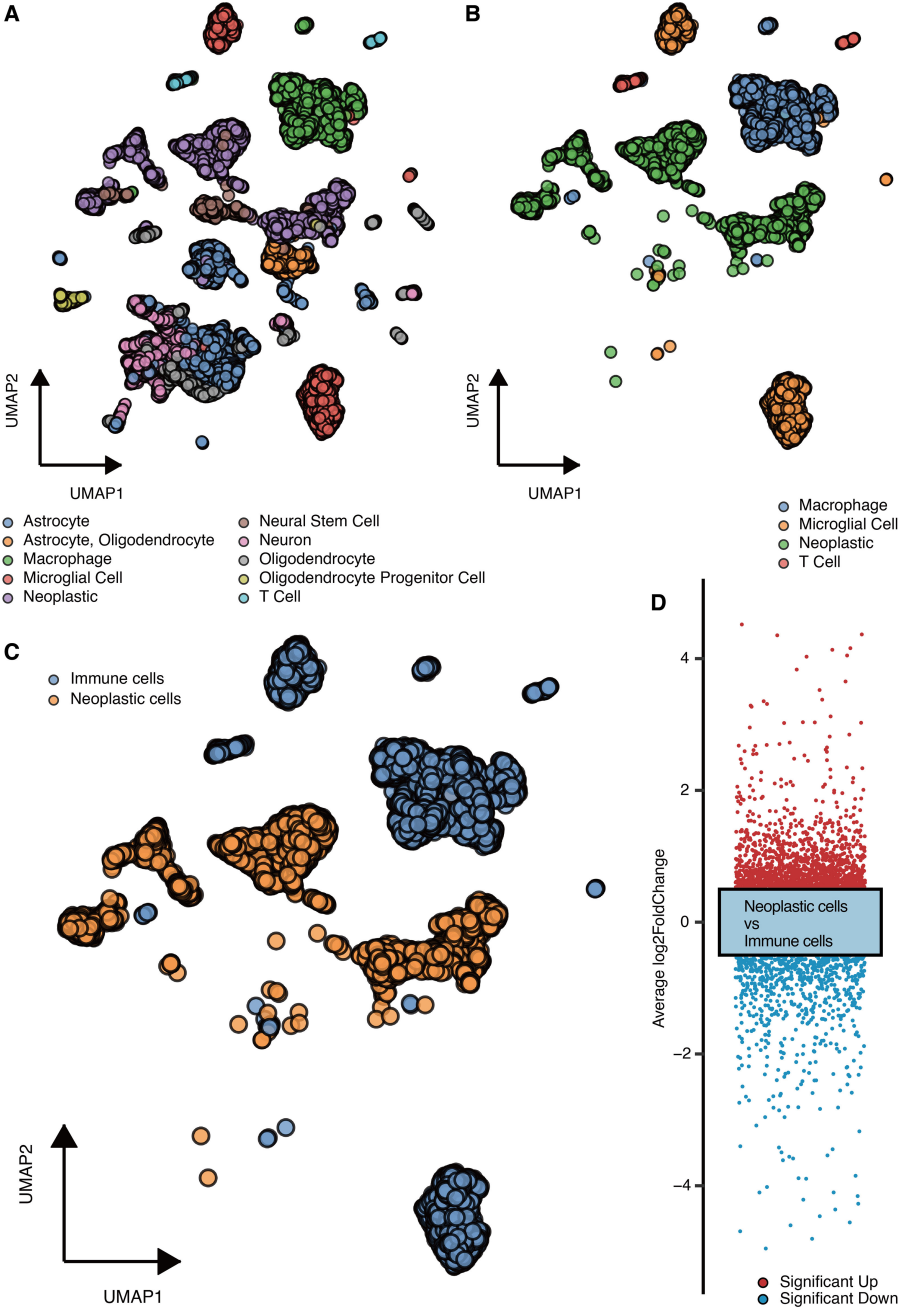
scRNA-seq analysis for malignant genes. **(A)** UMAP shows the microenvironment cells. **(B)** UMAP shows the major types of immune cells and neoplastic cells. **(C)** UMAP shows the immune cells and neoplastic cells. **(D)** DEGs between the immune cells and neoplastic cells.

The microenvironment cells (astrocyte, oligodendrocyte, macrophage, microglial cell, neoplastic, neural stem cell, neuron, T cell, etc.) in GBM are shown in **Figure 1A**. The major types of immune cells (macrophage, microglial cell, T cell) and neoplastic cells in GBM are shown in **Figure 1B**. The immune cells and neoplastic cells in GBM are shown in **Figures 1C, D**. DEGs between the immune cells and neoplastic cells are shown in **Figure 1D**.

### WGCNA for IE-related markers

**Figure 2:**
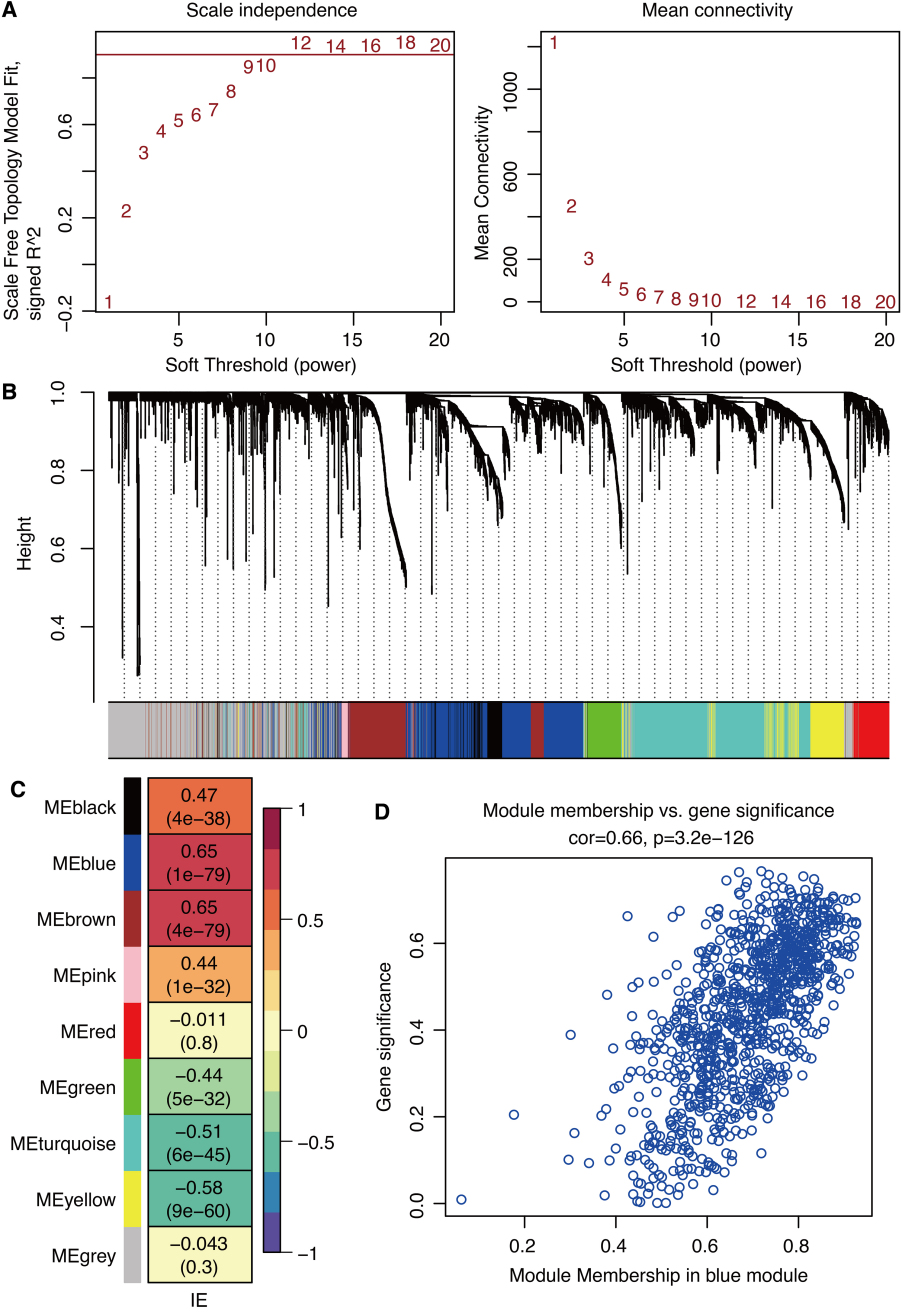
WGCNA for IE-related genes. **(A)** Scale-free topology model fit and mean connectivity. **(B)** Waterfall plot shows the gene models. **(C)** Correlation between gene modules and immune escape. **(D)** Correlation between gene significance and module membership.

ssGSEA was performed on IE pathway genes to calculate the IE score. Scale-free topology model fit and mean connectivity are shown in **Figure 2A**. WGCNA-based gene models in the glioma dataset are shown in **Figure 2B**. Correlation between gene modules and IE score showed that the blue module was the most correlated among the nine gene modules (**Figure 2C**). Gene significance is significantly associated with module membership in the blue module (**Figure 2D**).

### Machine learning for potent markers

**Figure 3:**
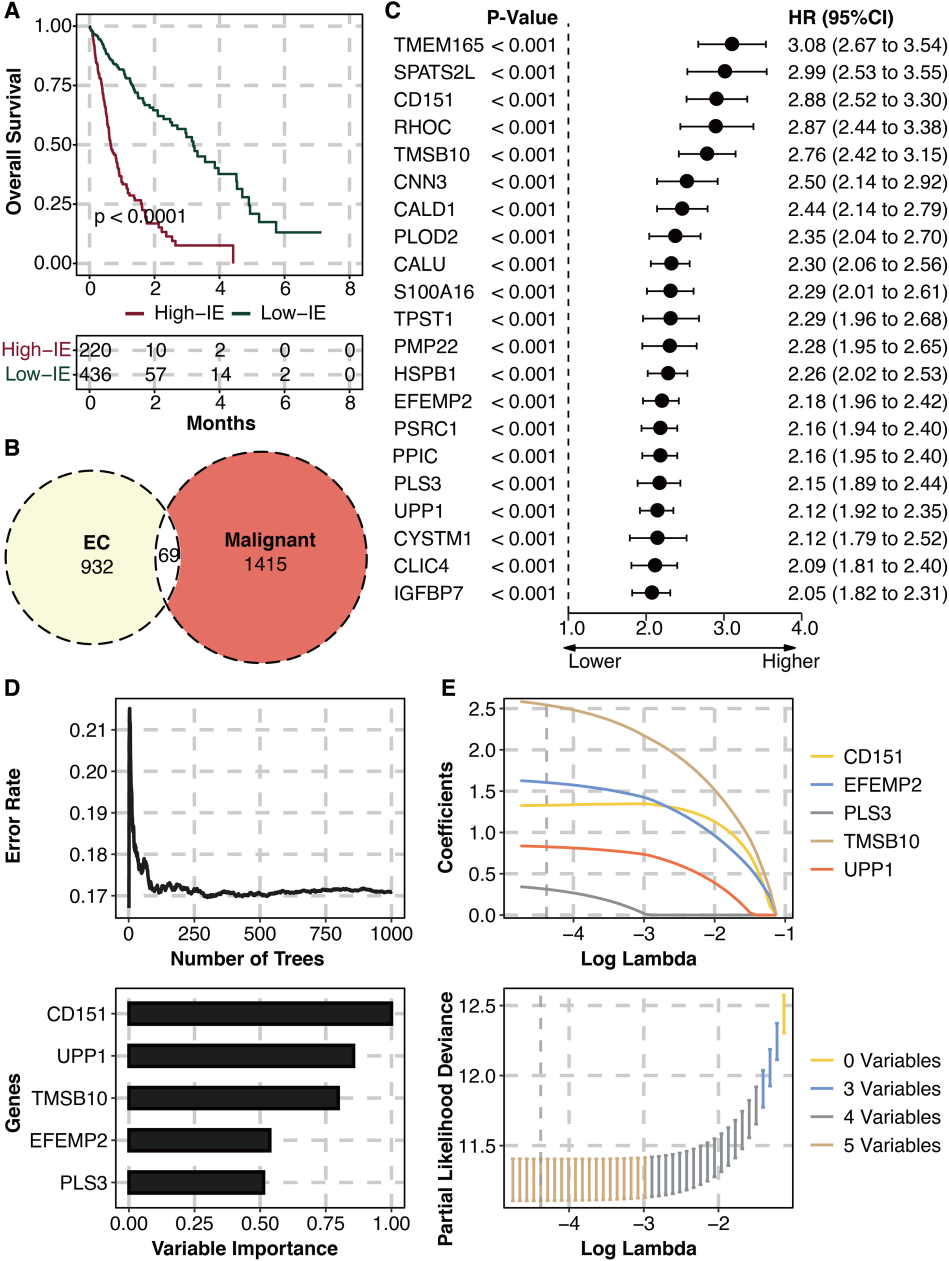
Machine learning for potent genes. **(A)** Survival plot shows the survival outcomes in the high and low IE groups. **(B)** Intersected genes between IE-related genes and malignant genes. **(C)** Univariate Cox regression analysis on intersected genes. **(D)** RSF analysis on prognostic intersected genes. **(E)** LASSO regression analysis on prognostic intersected genes.

The high IE group is related to worse survival (**Figure 3A**). 69 intersected genes between IE-related genes and malignant genes are identified (**Figure 3B**). Univariate Cox regression analysis on intersected genes showed that 21 genes were hazardous (**Figure 3C**). RSF analysis was performed for dimension reduction of prognostic intersected genes, which came to CD151, EFEMP2, PLS3, TMSB10, and UPP1 (**Figure 3D**). LASSO regression analysis was further performed for dimension reduction of prognostic intersected genes, which also came to CD151, EFEMP2, PLS3, TMSB10, and UPP1 (**Figure 3E**).

### Prognostic value of UPP1

**Figure 4:**
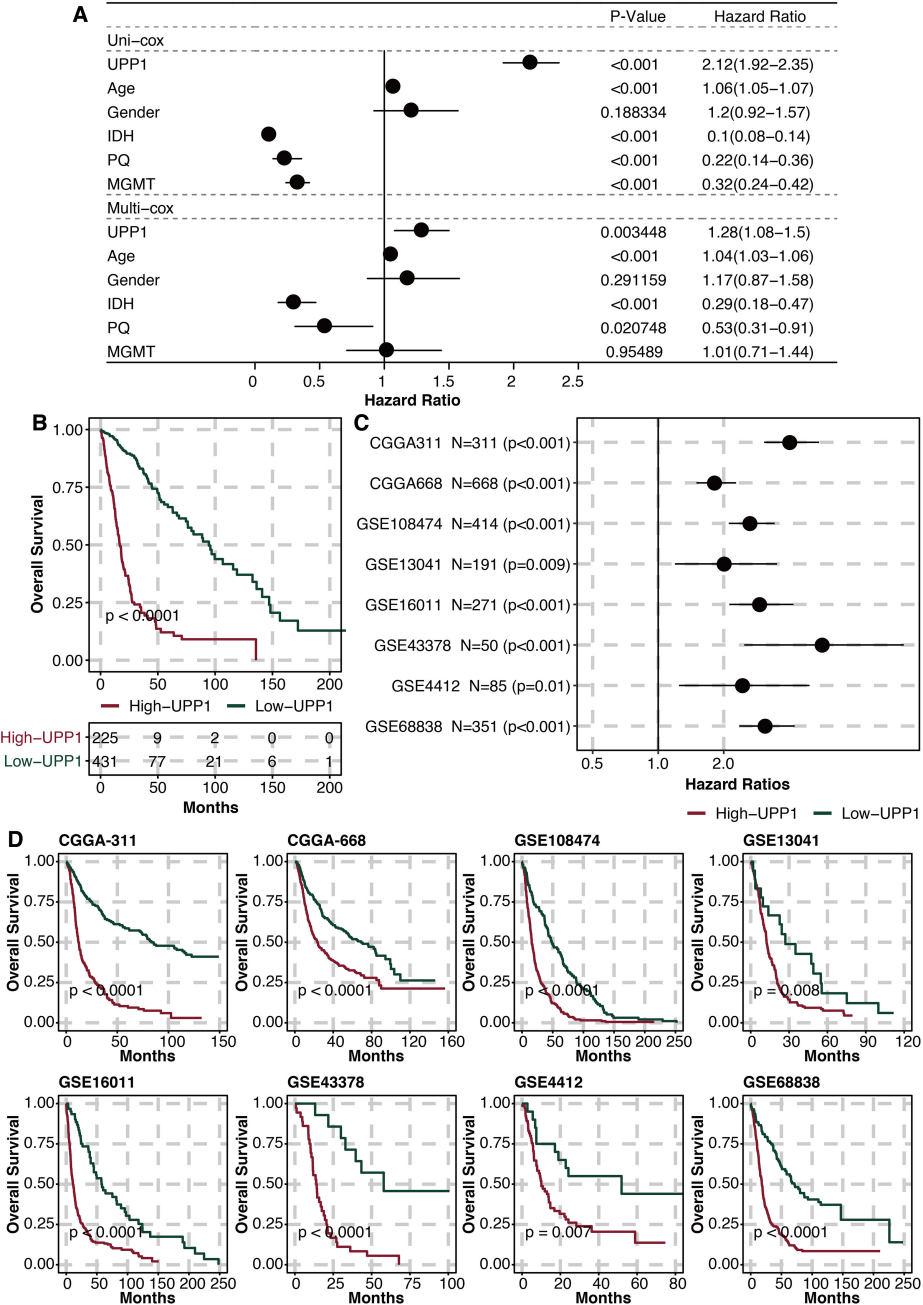
Prognostic value of UPP1. **(A)** Univariate and multivariate Cox regression analysis on UPP1 and clinical factors. **(B)** Survival plot shows the survival outcomes in the high and low UPP1 groups. **(C)** Univariate Cox regression analysis on UPP1 in different glioma datasets. **(D)** Survival plot shows the survival outcomes in the high and low UPP1 groups in different glioma datasets.

Univariate and multivariate Cox regression analysis on UPP1 and clinical factors (age, gender, IDH, 1p19q, MGMT) showed that UPP1 was an independent prognostic factor (**Figure 4A**). The high UPP1 group was related to worse survival (**Figure 4B**). Univariate Cox regression analysis on UPP1 in different glioma datasets showed that UPP1 was a hazardous marker (**Figure 4C**). The high UPP1 group was related to worse survival in different glioma datasets (**Figure 4D**).

### *In vitro* validation on UPP1

**Figure 5:**
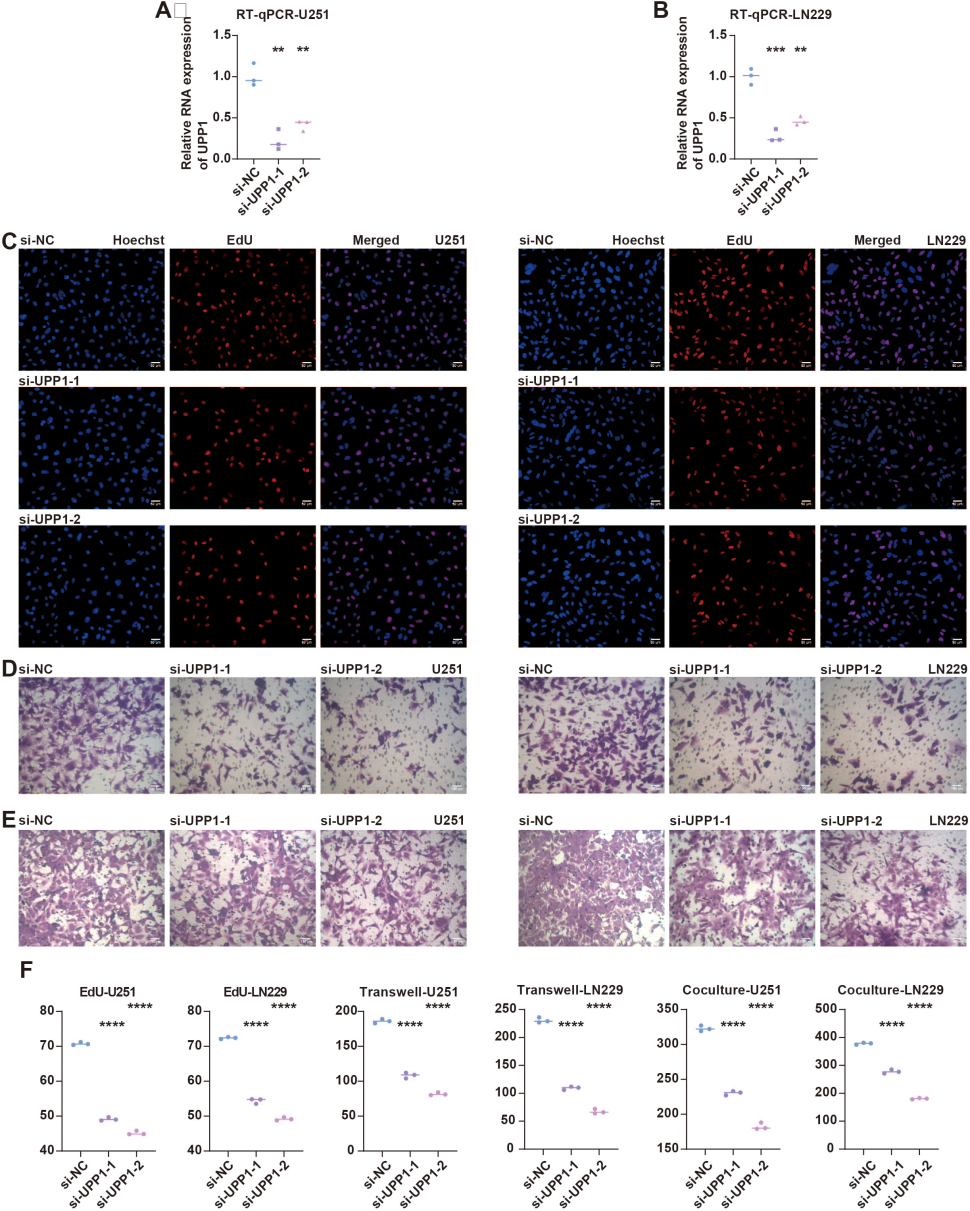
*In vitro* validation on UPP1. **(A)** RT-qPCR assay shows the RNA expression of UPP1 in different groups in U251 cells. **(B)** RT-qPCR assay shows the RNA expression of UPP1 in different groups in LN229 cells. **(C)** EdU assay shows the proliferated glioma cells in different groups in U251 and LN229 cells. **(D)** Transwell assay shows the migrated glioma cells in different groups in U251 and LN229 cells. **(E)** Co-culture Transwell assay shows the migrated macrophages in different groups in U251 and LN229 cells. **(F)** Statistical analysis of RT-qPCR, EdU, and Transwell assays in U251 cells. **(G)** Statistical analysis of RT-qPCR, EdU, and Transwell assays in LN229 cells. **, P < 0.01; ***, P < 0.001; ****, P < 0.0001.

Given the potential prognostic value of UPP1, experimental validation was performed. RT-qPCR assay showed that UPP1 expression was significantly suppressed in siRNA-transfected groups in U251 (**Figure 5A**) and LN229 (**Figure 5B**) cells. EdU assay showed that the proliferated glioma cells were significantly reduced in siRNA-transfected groups in U251 and LN229 cells (**Figures 5C, F**). Transwell assay shows the migrated glioma cells were significantly reduced in siRNA-transfected groups in U251 and LN229 cells (**Figures 5D, F**). Co-culture Transwell assay showed the migrated macrophages were significantly reduced in siRNA-transfected groups in U251 and LN229 cells (**Figures 5E, F**).

### Functional annotations of UPP1

**Figure 6:**
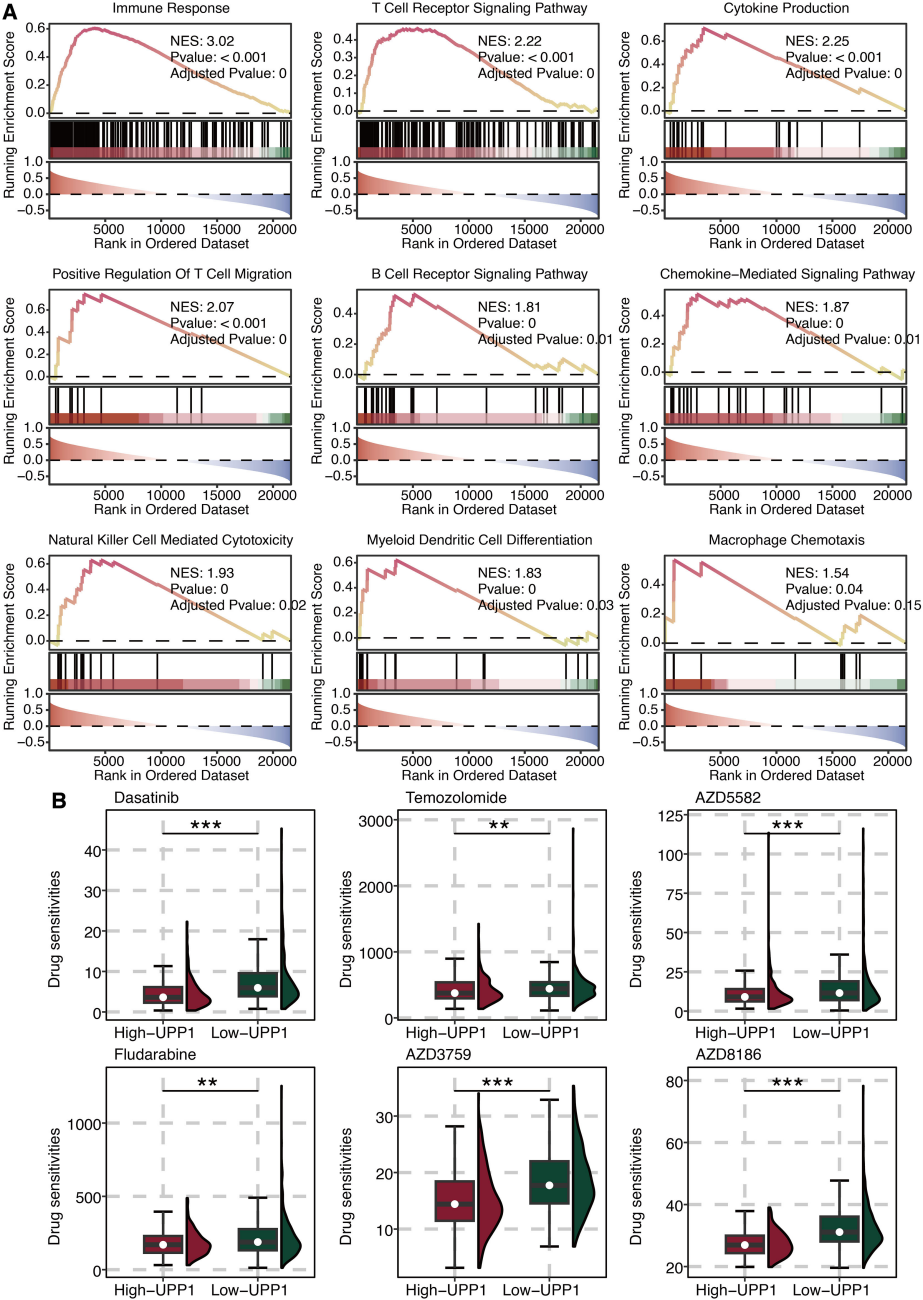
Functional annotation of UPP1. **(A)** GSEA on UPP1. **(B)** Drug prediction of UPP1. **, P < 0.01; ***, P < 0.001.

GSEA on UPP1 was performed, and immune pathways such as cytokine, chemokine, T cell activation, and macrophage activation were significantly enriched (**Figure 6A**). This indicates that UPP1 is intimately linked to the regulation of the tumor immune microenvironment. Drug prediction of UPP1 revealed that Dasatinib, Temozolomide, AZD5582, Fludarabine, AZD3759, and AZD8186 in the low UPP1 group had significantly higher drug sensitivity (**Figure 6B**).

### Immunological features of UPP1

**Figure 7:**
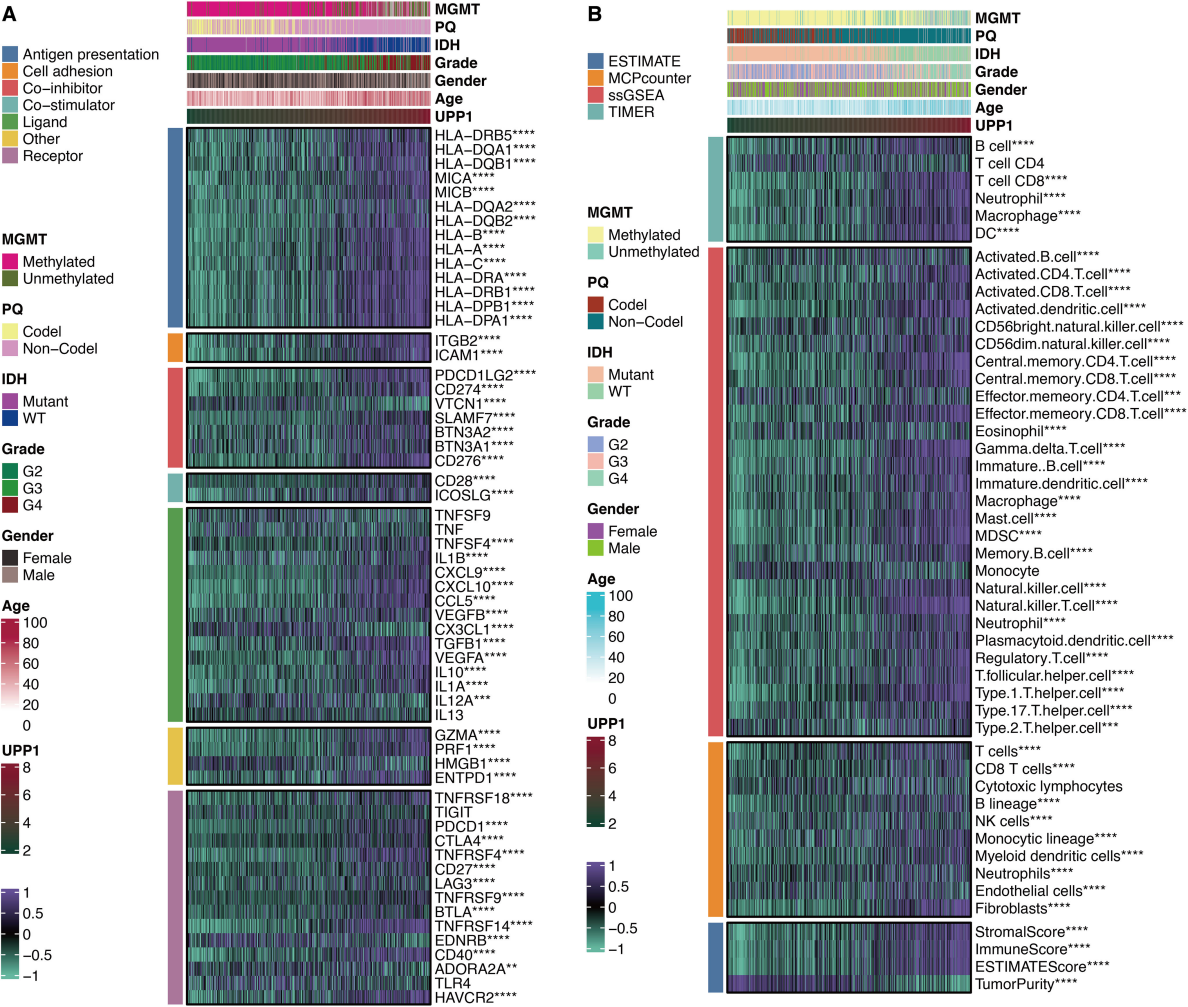
Immune features of UPP1. **(A)** Correlation between UPP1 and immune modulators. **(B)** Correlation between UPP1 and immune cells. **, P < 0.01; ***, P < 0.001; ****, P < 0.0001.

UPP1 was significantly associated with immune modulators CD274, CD276, CD28, and ICOSLG (**Figure 7A**). This suggests that UPP1 may contribute to immune evasion by modulating the expression of these immune checkpoint molecules. Besides, UPP1 was significantly associated with immune cells DCs, B cells, T cells, MDSCs, Tregs, and macrophages (**Figure 7B**). This indicates that UPP1 may play a role in shaping the composition and function of the tumor immune microenvironment.

### Immunotherapy response prediction of UPP1

**Figure 8:**
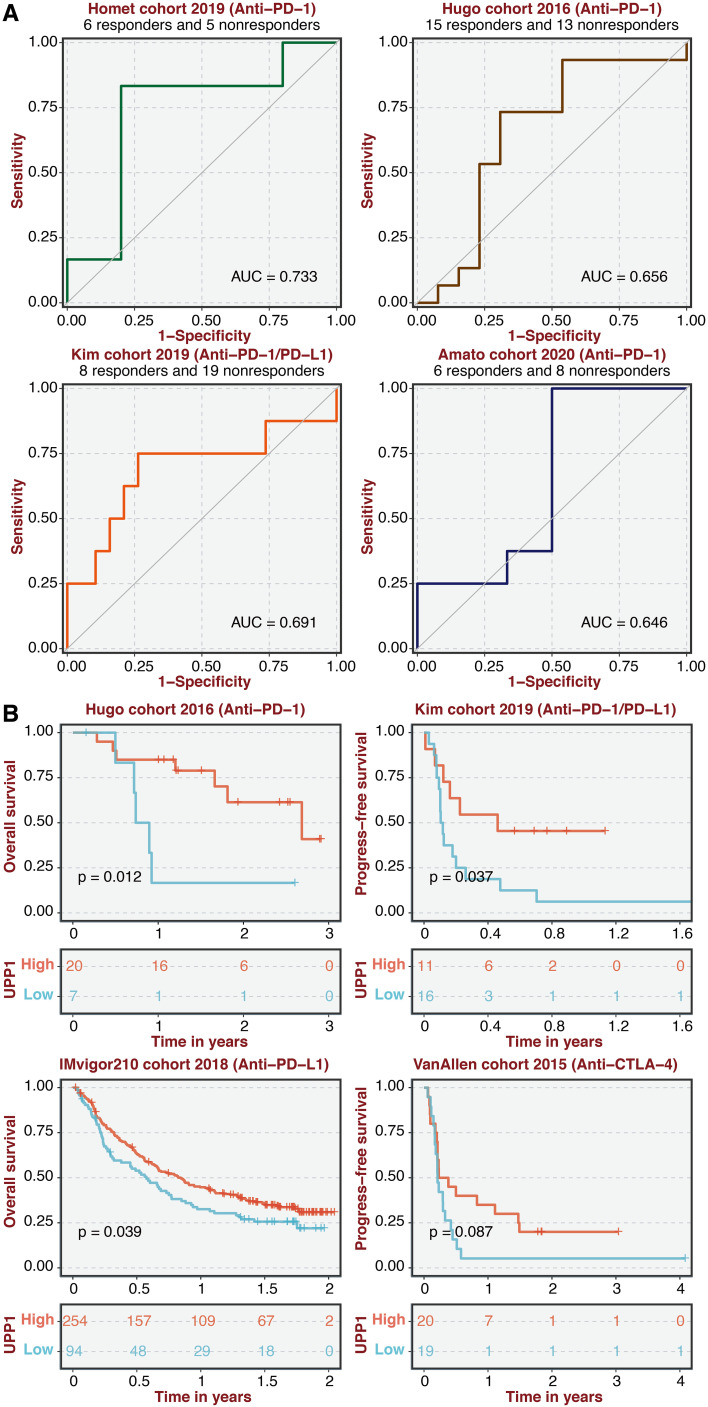
Immunotherapy prediction of UPP1. **(A)** ROC curves of UPP1 in four immunotherapy cohorts. **(B)** Survival plot shows the survival outcomes in the high and low UPP1 groups in four immunotherapy cohorts.

ROC curves of UPP1 in four immunotherapy cohorts showed that UPP1 could effectively predict immunotherapy responses (**Figure 8A**). Besides, the high UPP1 group was associated with better survival in four immunotherapy cohorts (**Figure 8B**).

### Mutation characteristics of UPP1

**Figure 9:**
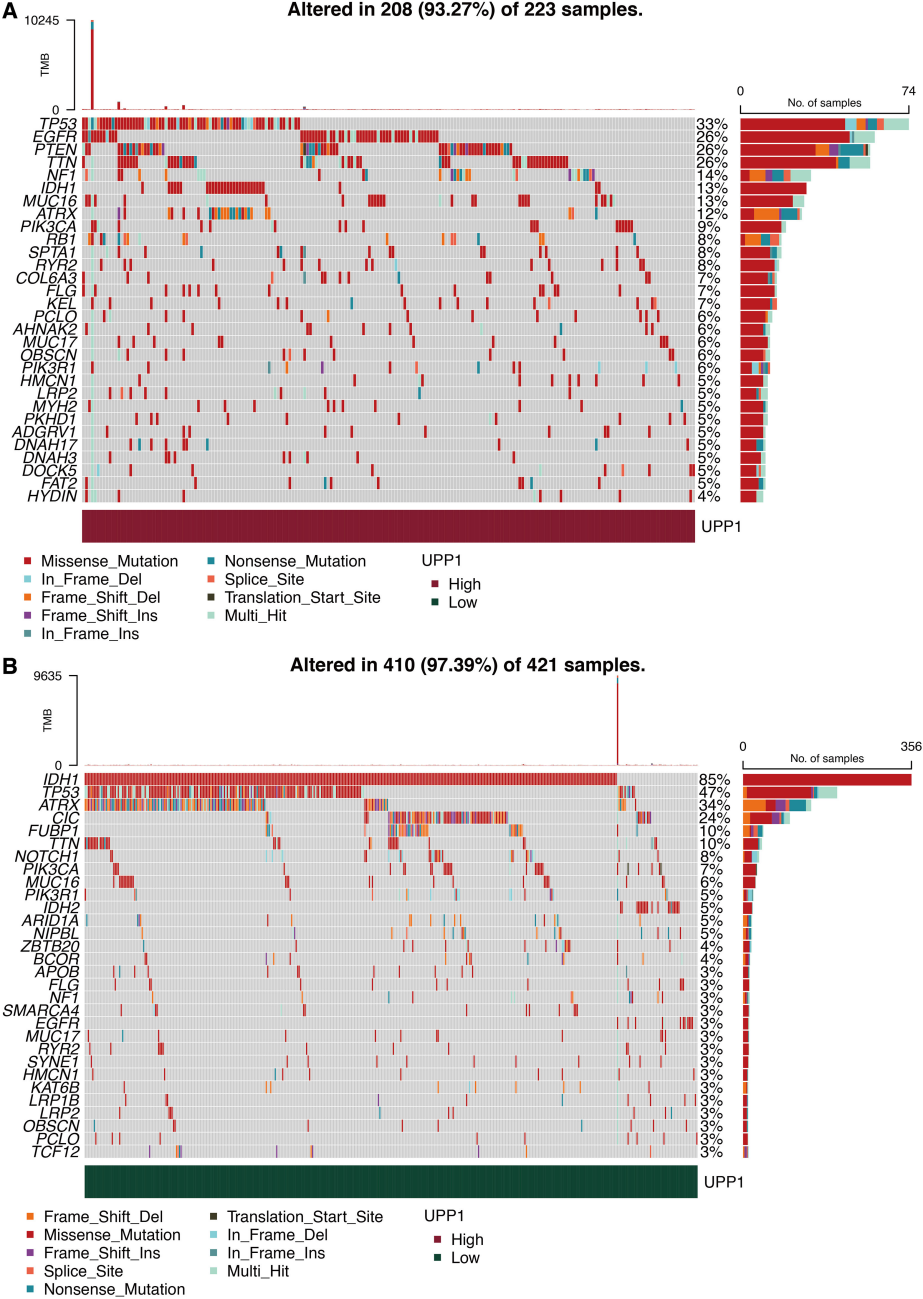
Mutation characteristics of UPP1. **(A)** Mutation landscape in high UPP1 group. **(B)** Mutation landscape in low UPP1 group.

The mutation landscape in the high UPP1 group is shown in **Figure 9A**, in which EGFR and PTEN were highly mutated. The mutation landscape in the low UPP1 group is shown in **Figure 9B**, in which TP53 and IDH were highly mutated. Differentially expressed mutation genes in the high and low UPP1 groups are shown in **Supplementary Figure 1A**, in which IDH was the top-ranked mutated gene in the low UPP1 group. Mutually mutated gene pairs in the high UPP1 group are shown in **Supplementary Figure 1B**. Mutually mutated gene pairs in the low UPP1 group are shown in **Supplementary Figure 1C**.

### Pan-cancer analysis of UPP1

UPP1 expression was significantly higher in tumor and normal tissues in most cancer types (**Supplementary Figure 2A**). Univariate Cox regression analysis of UPP1 confirmed that UPP1 was a hazardous marker in most cancer types (**Supplementary Figure 2B**).

## Discussion

The rapid advancement of high-throughput genomic and molecular profiling technologies has generated vast amounts of complex biological data in cancer research. This data deluge has necessitated the development of sophisticated computational approaches to extract meaningful insights and patterns from the data (19). Machine learning, a field of artificial intelligence, has emerged as a powerful tool to tackle these challenges in cancer research. Machine learning models can analyze multi-omics data, such as genomics, transcriptomics, and proteomics, to identify robust molecular biomarkers predictive of cancer risk, prognosis, or treatment response (20). This can help guide the development of personalized cancer diagnostics and therapeutics. Our integrative analysis of single-cell and bulk tumor sequencing data by machine learning identified the gene UPP1 as a critical driver of glioma tumorigenesis and immune evasion. UPP1 encodes the enzyme uridine phosphorylase 1, which catalyzes the reversible phosphorolysis of uridine and 2’-deoxyuridine (21). It plays a significant role in the ubiquitin-proteasome system, which is essential for maintaining cellular homeostasis by regulating protein turnover. In the context of cancer, UPP1 is involved in various aspects of cancer development and progression, including regulation of protein homeostasis, cell cycle regulation, apoptosis and survival, angiogenesis and metastasis, and immune evasion (22).

Elevated expression of UPP1 was associated with significantly worse patient survival across multiple independent glioma cohorts. Functional studies demonstrated that silencing UPP1 in glioma cell lines reduced tumor cell proliferation and invasion, enhancing anti-tumor immune responses through increased cell recruitment and activation of macrophages. These results indicate that UPP1 plays a dual role in promoting intrinsic tumor growth and immunosuppression within the glioma microenvironment. UPP1 plays a significant role in modulating macrophage activity through several potential mechanisms: UPP1 is involved in the ubiquitin-proteasome pathway, where it tags dysfunctional proteins for degradation. By regulating protein turnover, UPP1 helps maintain macrophage homeostasis, ensuring that only functional proteins are present for critical immune responses. UPP1 can influence the production of pro-inflammatory cytokines. By degrading specific proteins involved in inflammatory signaling pathways, UPP1 may help fine-tune the macrophage response to pathogens and tissue damage, preventing excessive inflammation that could lead to tissue injury. Macrophages play a crucial role in antigen presentation. UPP1 may facilitate the processing of antigens by regulating the degradation of precursor proteins, thus enhancing the ability of macrophages to present antigens to T cells and initiate adaptive immune responses. UPP1 can affect the expression of surface receptors involved in phagocytosis. By regulating the turnover of these receptors, UPP1 may enhance or diminish the macrophage’s ability to engulf and eliminate pathogens or debris. In response to environmental stress, UPP1 can help macrophages adapt by managing the levels of proteins involved in stress responses. This may enhance macrophage survival and functionality under adverse conditions, such as during infection or inflammation. UPP1 may interact with various signaling pathways, such as NF-kB and MAPK pathways, which are critical for macrophage activation and function. By modulating these pathways, UPP1 can influence macrophage differentiation, activation, and effector functions.

The identification of UPP1 as a driver of glioma malignancy is notable, as the role of this enzyme in cancer pathogenesis has been relatively unexplored. Previous studies have primarily focused on the potential utility of UPP1 as a target for cancer chemotherapy, given its involvement in the metabolism of nucleoside analogs (23, 24). Our findings suggest a more fundamental role for UPP1 in regulating core tumorigenic processes, including cell proliferation, migration, and immune evasion.

Mechanistically, UPP1 may promote glioma progression through several potential pathways. At the metabolic level, UPP1-mediated catabolism of nucleosides could influence nucleotide biosynthesis, DNA repair, and other proliferation-associated processes (25). UPP1 has also been linked to regulating inflammatory signaling cascades, which could modulate the anti-tumor immune response (26, 27). Through the PI3K/AKT/mTOR pathway, UPP1 overexpression also increases the production of PD-L1, which aids in inhibiting CD8+ T cells and shapes the immunosuppressive nature of the TME (27). Further investigation is needed to fully elucidate the downstream effectors of UPP1 that drive its pro-tumorigenic and immunosuppressive functions. It is hypothesized that PP1’s role in nucleotide metabolism allows cancer cells to adapt their energy production and biosynthetic pathways, enhancing their survival and competitive advantage in nutrient-poor environments.

In addition to its prognostic significance, our analysis indicates that UPP1 expression levels could be a useful biomarker to predict other clinically relevant tumor characteristics. High UPP1 was associated with specific genomic alterations, drug response profiles, and immune infiltration patterns. The differential mutation patterns observed in the high UPP1 and low UPP1 groups may provide insights into the potential mechanisms by which UPP1 expression influences cancer biology. For example, the interplay between UPP1 and the deubiquitination of key oncogenic or tumor suppressor proteins, such as those encoded by EGFR, PTEN, TP53, and IDH, may be an important factor in cancer development (28). This suggests that UPP1 could be a versatile marker to help guide personalized treatment approaches for glioma patients. Notably, the expression level of UPP1 could be used as a biomarker to predict the sensitivity of cancer cells to certain drugs, such as Dasatinib, Temozolomide, AZD5582, Fludarabine, AZD3759, and AZD8186. Understanding the relationship between UPP1 expression and drug sensitivity could help develop personalized treatment strategies where the choice of drug therapy is based on the UPP1 status of the cancer (29, 30). In addition, the ability of UPP1 to predict immunotherapy responses and its association with better survival in immunotherapy-treated patients suggest a complex interplay between UPP1 and anti-tumor immunity. While high UPP1 expression was generally associated with immune suppression, the improved outcomes in the immunotherapy cohorts indicate that the heightened immune response elicited by immunotherapy can overcome the suppressive effects of UPP1.

In conclusion, our study has uncovered a previously unappreciated role for the metabolic enzyme UPP1 as a driver of glioma malignancy. Targeting UPP1 or its downstream effectors may represent a promising therapeutic strategy for this devastating disease. Further research is needed to elucidate the precise molecular mechanisms by which UPP1 drives tumor progression and immune evasion. Investigating the downstream signaling pathways and regulatory networks of UPP1 could uncover additional therapeutic vulnerabilities. Besides, the role of UPP1 in tumorigenesis and immune evasion identified in this study may not be limited to glioma. Investigating the potential implications of UPP1 in other cancer types could uncover broader therapeutic applications. There are also some limitations of the study. While UPP1 shows promise as a therapeutic target, the study does not provide a detailed analysis of potential resistance mechanisms or how targeting UPP1 could interact with existing therapies. Besides, a real-world cohort is expected to confirm the prognostic roles of UPP1.

## Data Availability

The original contributions presented in the study are included in the article/**Supplementary Material**. Further inquiries can be directed to the corresponding authors.

## Supplementary material

Supplementary Figure 1 Mutation characteristics of UPP1. **(A)** Differentially expressed mutation genes in the high and low UPP1 groups. **(B)** Mutually mutated gene pairs in high UPP1 group. **(C)** Mutually mutated gene pairs in low UPP1 group.

Supplementary Figure 2 Pan-cancer analysis of UPP1. **(A)** Vlnplot shows the expression of UPP1 in tumor and normal tissues in pan-cancer. **(B)** Univariate Cox regression analysis of UPP1 in pan-cancer.
