# Supplementary figures and images for: Machine learning-based discovery of UPP1 as a key oncogene in tumorigenesis and immune escape in gliomas
Source: Front Immunol. 2024 Sep 24;15:1475206. doi: 10.3389/fimmu.2024.1475206 (PMC11458454; doi:10.3389/fimmu.2024.1475206)

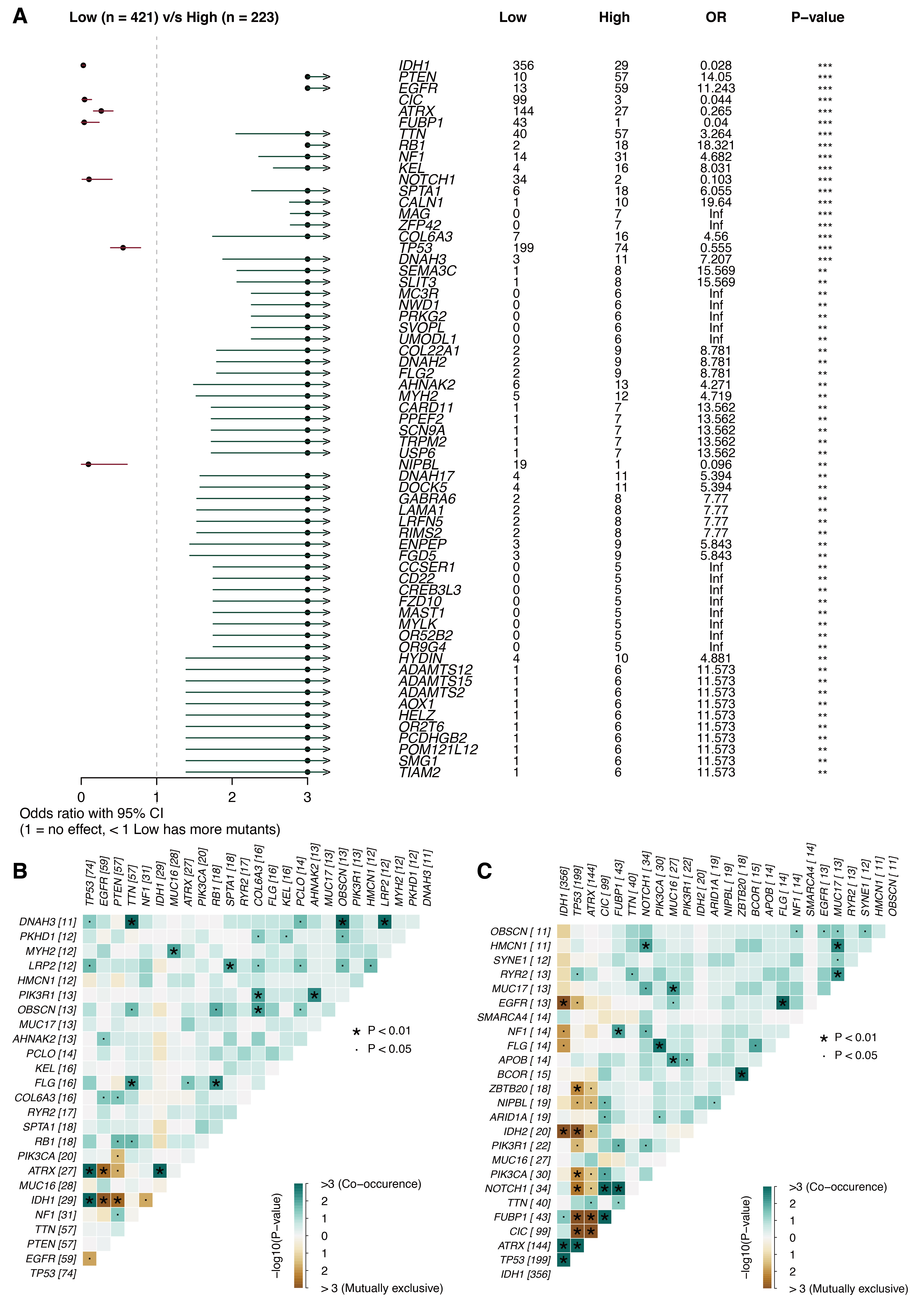

Supplement: Supplementary Figure 1 — Mutation characteristics of UPP1. (A) Differentially expressed mutation genes in the high and low UPP1 groups. (B) Mutually mutated gene pairs in high UPP1 group. (C) Mutually mutated gene pairs in low UPP1 group. [file Image1.jpeg]

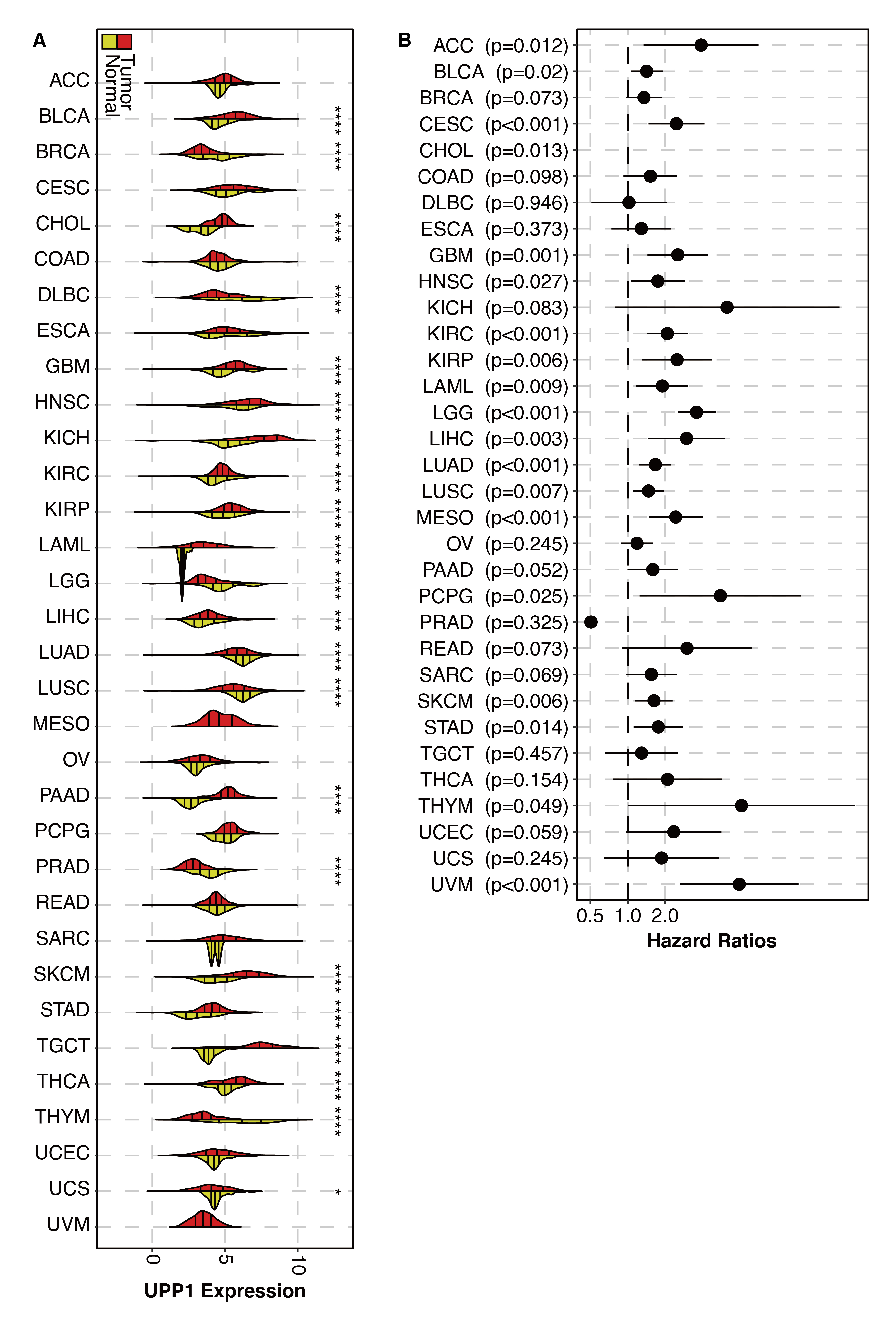

Supplement: Supplementary Figure 2 — Pan-cancer analysis of UPP1. (A) Vlnplot shows the expression of UPP1 in tumor and normal tissues in pan-cancer. (B) Univariate Cox regression analysis of UPP1 in pan-cancer. [file Image2.jpeg]
